# Pacemaker lead endocarditis with hiccups (Kalayci)

**DOI:** 10.22088/cjim.9.3.299

**Published:** 2018

**Authors:** Belma Kalaycı, Turgut Karabag, Turgay Erten, Tunahan Akgun

**Affiliations:** 1 Bulent Ecevit University, School of Medicine, Department of Cardiology, Zonguldak, Turkey

**Keywords:** Hiccup, Pacemaker lead, Infective endocarditis, Echocardiography

## Abstract

**Background:**

Lead-related infections that might develop after pacemaker implantation associated with high mortality and morbidity rates are challenging to manage and pose high-cost. Patients with lead-related infections usually present with fever, chills and fatigue and the treatment can be challenging unless the implant system is extracted.

**Case presentation:**

A 66-year old male patient who underwent dual chamber pacemaker and implantable cardioverter defibrillator was admitted to the emergency service with a six-week history of complaints of hiccups and fever. After a detailed investigation, lead-related infective endocarditis was the diagnosis. The patient was initiated on antibiotic therapy and lead extraction was performed.

**Conclusions:**

Patients with signs of infection who underwent pacemaker implantation may present with atypical symptoms such as hiccup. In these cases, imaging, particularly echocardiography, should be performed as soon as possible and the localization of the pacemaker leads and signs of infective endocarditis should be investigated.

Lead-related infective endocarditis (LRIE) is a serious disease and have increased in proportion with the increase in implantation rate (1-4), the main reasons of which include the increasingly frequent performance of implantation procedures and the performance of CIED procedures-mostly in the elderly population with higher comorbidity. In addition, procedure related factors should not be ignored (3). In the case of the development of lead related infective endocarditis (IE), it would be difficult to treat infection without removing the system (5). Patients with lead-related infection may present with nonspecific signs including fever, chills, and sweating. This study demonstrated a case of IE with vegetation on the tricuspid valve and pacemaker lead in a 66 year-old male patient with hiccup which is an extremely rare symptom who had underwent permanent pacemaker implantation three months ago.

## Case Presentation

A 66-year old male patient was admitted to the emergency service with a six-week history of complaints of hiccup and fever. Hiccups mostly correlated with lead stimulation. The patient had a diagnosis of dilated cardiomyopathy and underwent dual chamber pacemaker and implantable cardioverter defibrillator implantation (ICD) due to atrioventricular complete block three months ago in another hospital. A coronary angiography was performed before the procedure showed normal coronary arteries. Six weeks after the implantation, the patient had fever and was given antibiotic therapy in another hospital. The medical history revealed diabetes and hypertension for 10 years. 

**Figure 1:**
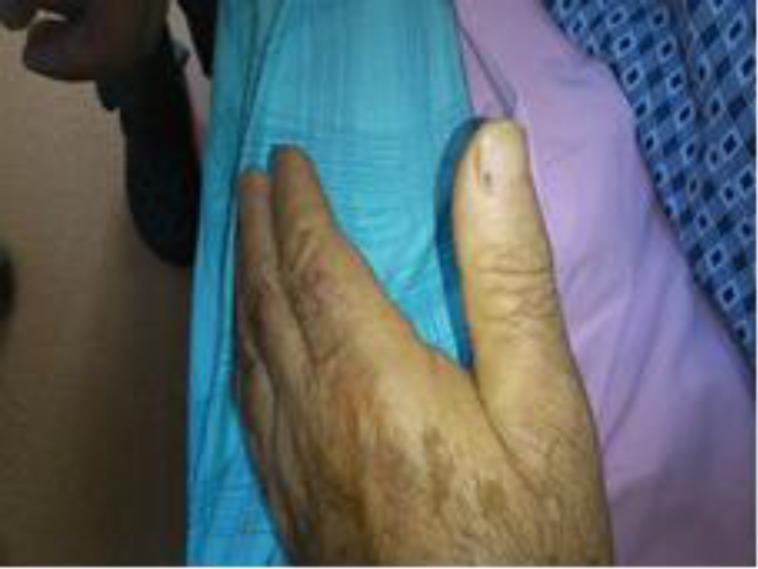
Splinter hemorrhages of the patient with infective endocarditis

On admission, the patient had a fever of 39.5°C, blood pressure of 100/50 mmHg and heart rate of 100/min. Pulmonary sounds were rough and there was no crepitation. A 2/6 pansystolic murmur was audible at all cardiac foci. There was mild edema in both legs. There were splinter hemorrhages in the nails (figure 1). 

**Figure 2:**
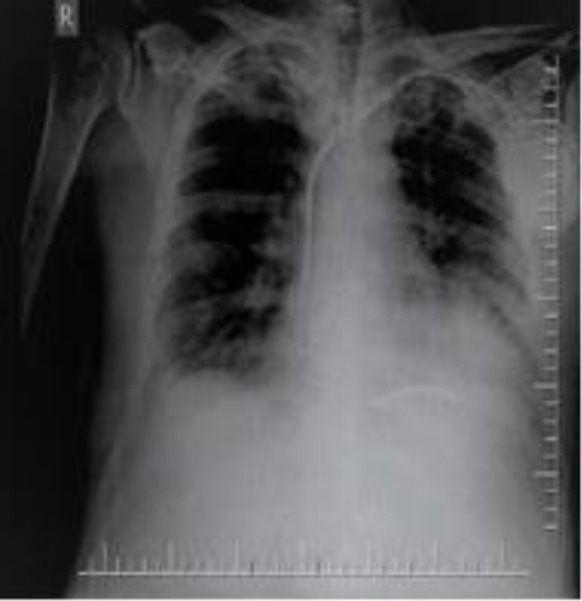
Implantable cardioverter defibrillator, increased cardiothoracic ratio, bilateral pleural effusions, and dual chamber pacemaker on chest x-ray.

An electrocardiogram showed pacemaker rhythms. A bigeminy ventricular extrasystole was noted. A chest x-ray revealed a bilateral reticulonodular pattern. There were bilateral pleural effusions. The cardiothoracic index was increased. The pacemaker generator implanted in the left pectoral region was present along with two (atrial and ventricular) leads, which appeared to extend into the heart (figure 2). 

**Figure 3:**
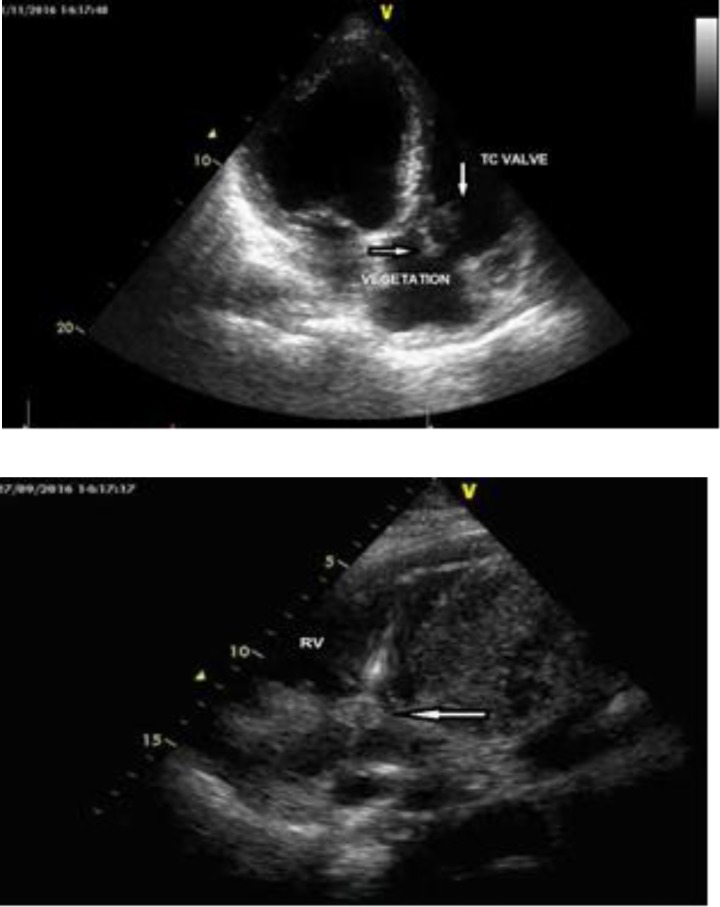
Vegetations floating freely beneath the tricuspid valve (A) and wrapped around the lead and obstructing the tricuspid orifice (B) on apical imaging. (TC; tricuspid, RV; right ventricle)

Laboratory tests revealed a WBC of 12.300/m3, sedimentation of 62mm, and CRP of 235 mg/L. HbA1C level was 6.2%. The patient with these clinical manifestations was hospitalized with the diagnosis of unknown fever and hiccups in the department of infectious diseases and was initiated on drug therapy with sulbactam+ampicillin+ciprofloxacin. His blood culture yielded positive result for methicillin-sensitive staphylococcus aureus. His body temperature tended to decrease for a while but increased again to 39°C on day nine, upon which the patient was designated to undergo transthoracic echocardiography. The hiccups continued with intervals. A transthoracic echocardiography showed global hypokinesia of the left ventricle with a low ejection fraction (EF= %35). Besides mild mitral insufficiency, the patient had moderate tricuspid insufficiency. There was an amorphous and mobile mass measuring 2x2.1 cm that was consistent with vegetation, both floating freely on the atrial surface of the tricuspid valve and attached to the pacemaker lead and was continuous inside the ventricle. The mass appeared to be obstructing the tricuspid orifice intermittently (figures 3A, B). 

These findings confirmed the diagnosis of lead-related infective endocarditis. Given the presence of resistant fever and the size of the mass, lead extraction was planned. The patient was initiated on antibiotic therapy with sulbactam+ampicillin+rifampicin. Upon his request, the patient was transferred to the center where the pacemaker had been implanted. In the mentioned center, the pacemaker lead was extracted and implanted as a temporary transvenous pacemaker. Antibiotic therapy was given for two weeks. After the signs of infection disappeared, a dual chamber pacemaker and ICD were implanted in the patient. After implantation, antibiotic treatment was continued for two weeks. The patient who was followed-up 3 months later was in pacemaker rhythm. He did not complain hiccups, was in good condition and had no signs of infection. There was no echocardiography vegetation. 

## Discussion

The infection rate in patients with permanent pacemaker implant was found to be 0.4– 16.3 %, whereas lead- related IE was detected in less than 10 % of cases (4). These infections remain a major risk on patients and whether it is treated or not, pose an important problem because of the associated high economic burden on the health system (6). The annual mortality is 20% in those in whom the device was extracted and 38% with device left in place (7). Staphylococcus aureus is the most common pathogen of these infections, whereas, coagulase negative staphylococcus is also a common cause. Coagulase negative staphylococci tend to cause a larger vegetation (8). 

Patients with lead-related infection may present signs of infection including fever, chills, and sweating. These clinical signs may be caused by local infections at the site of the pacemaker pocket or patients may manifest a range of serious clinical signs from bacteremia to sepsis. Celik et al. reported two cases presented with hiccup in patients with lead perforation. They concluded mechanical effect of perforated lead in one patient, phrenic nerve stimulation by the lead in another patient as the causes of hiccups (9). The displacement of the lead in particular retraction into the brachiocephalic vein, at the level of esophagus and anatomic proximity to both recurrent or phrenic nerves may cause an electrical irritation of these nerves (9-10). Gasparini et al. reported a case presented with hiccups in a patient with Twiddler’s syndrome (9). In our case; atrial and ventricular leads appear to be in correct anatomical position and there was no Twiddler’s syndrome. The hiccups were mostly correlated with lead stimulation. We consider that hiccups developed secondary to phrenic nerve irritation as a result of lead vegetation on the position of lead.

The diagnosis of lead-related IE is made by the visualization of lead vegetation on echocardiography and the documentation of infection in blood (8). Intravenous drug use, structural heart diseases including ventricular septal defect, prosthetic valve disease, rheumatoid valve disease, previous infective endocarditis, hemodialysis, and immunocompromised conditions are the predisposing factors. In the presence of lead-related infection, it is quite difficult to cure infection without total extraction of the system (4).

In the case presented here, clinical signs of infection developed shortly after pacemaker implantation, indicate that procedure-related factors were also effective. Our patient had no predisposing factors that might have caused infective endocarditis such as intravenous drug use, hemodialysis, or structural heart disease.

 Despite the initiation of staphylococcus aureus antibiotic sensitivity in the patient, the infection could not be controlled. The extraction of the implanted system at the earliest in these patients who developed drug-resistant infection particularly would be a better option. In our institute, we do not have the facility for lead extraction. So we had to transfer the patient to another hospital. This situation was the main weakness of the presented case. It is interesting that despite the absence of lead perforation or lead dislocation in our case, the patient presented with resistant hiccup. We consider that hiccups developed secondary to phrenic nerve irritation as a result of the effect of lead vegetation on the lead position. The fact is that the correlation of hiccups with lead stimulation supported our conclusion.

In conclusion, pacemakers implantations have become more widespread in use so as lead-related infections. To prevent infections, performing the procedures in a sterilized fashion and administering antibiotic prophylaxis are of great significance, about which physicians and healthcare providers should be particular and take necessary precautions. Patients with signs of infection who underwent pacemaker implantation may present with atypical symptoms such as hiccup. In these cases, imaging, particularly echocardiography, should be performed as soon as possible and the localization of the pacemaker leads and signs of infective endocarditis should be investigated. 

## Funding:

No funding has been allocated for this study.

## Conflict of interest:

None declared
